# Predictive Shapes of Ellipsoid PPDL-PTHF Copolymer Particles Prepared by the Phantom Stretching Technique

**DOI:** 10.3390/polym14183762

**Published:** 2022-09-08

**Authors:** Christian Wischke, Dieter Hofmann

**Affiliations:** Institute of Active Polymers, Helmholtz-Zentrum Hereon, Kantstrasse 55, 14513 Teltow, Germany

**Keywords:** prolate ellipsoids, shape prediction model, aspect ratio, microparticle shape

## Abstract

Ellipsoidal polymer particles can be prepared from spheres by unidirectional stretching at elevated temperatures, while the particles’ aspect ratios (*AR*) that result from this phantom stretching methodology are often not precisely predictable. Here, an elastic deformation model was exemplarily evaluated for ~50 µm spherical microparticles from PPDL-PTHF block copolymers. The prolate ellipsoidal particles, obtained by stretching in polyvinyl alcohol phantoms, differed in dimensions at identical relative phantoms elongations up to 150%, depending on the relative polymer composition and their systematically altered mechanical properties. Importantly, the resulting particle shapes within the studied range of *AR* up to ~4 matched the predictions of the elastic deformation model, which includes information of the elastic moduli of phantom and particle materials. These data suggest that the model may be applicable to predict the conditions needed to precisely prepare ellipsoids of desired *AR* and may be applicable to various deformable particle materials.

## 1. Introduction

The interest in non-spherical particles, such as ellipsoids, spans over various disciplines, including geology [1], physics [2], material sciences [3] and pharmaceutics [4,5]. In life sciences, some fundamental observations on the effects of particle shape on phagocytosis [6] and the interaction with model endothelial substrates under shear flow [7] motivated excessive studies on shape-dependency of biodistribution [8,9], drug delivery [10], and ecotoxicity [11].

For both, model studies and potential applications of non-spherical particles, their precise and reproducible production is a key element [12]. Accordingly, a substantial number of concepts were explored to obtain non-spherical particles [13], e.g., by subjecting suspensions of deformable spheres to shear forces with subsequent shape fixation [14,15], ion beam deformation of silica spheres [16], deposition of various classes of materials in cavities of deformable polymeric foams [17], UV crosslinking of polymers in microfluidic set-ups [18,19], or imprinting techniques [20]. All of these techniques have prerequisites that may impede their applicability to various types of materials. Specifically, many methods are restricted by the fact that direct comparability of the anisotropic particles cannot be achieved, as they do not originate from the same stock particles/materials.

In contrast, the phantom deformation technique, first patented in the late 1980’s [21], holds the great advantage that the shape of preformed stock particles can be modified. The principle of this technique is to temporarily embed the particles into another material, a deformable matrix, which allows simultaneous deformation of the particles by phantom stretching [22]. More recently, the Mitragotri group, and subsequently many other researchers, have used the concept to design various particle shapes from preformed polymer spheres by phantom stretching or compression [3,6,13,23]. Preferentially, polyvinyl alcohol (PVA) of low molecular weight and a degree of deacetylation of 80–90% is used as the phantom matrix material, as it can be dissolved in water, allowing the collection of the deformed particles. However, a major challenge that remains in using the phantom stretching technique for various types of incorporated particles involves obtaining the desired aspect ratios *AR* in a reproducible and predictable manner [24].

In order to tackle the issue of producing predictable and reproducible shapes, the applicable frame conditions for successful deformation of embedded particles should be taken into account. It is obvious that, for example, hard metal particles will not be deformed when the PVA phantom is stretched at common temperatures of 60 to 100 °C. In contrast, particle deformation should be successful when they are relatively soft at the conditions of phantom deformation. In order to achieve softness of the particles, elevated temperatures or solvent-induced plasticization may be used.

Given the relevance of the phantom stretching technique for several types of particulate materials, it would be useful to demonstrate the suitability of a model [25] to predict particle shapes at different degrees of phantom stretching by considering the effect of the materials’ mechanical properties. In this study, a set of multiblock copolymers with variations in block content, and thus a variation in their elastic modulus *E,* was used to prepare spherical microparticles as model stock materials. These particles were deformed via PVA phantom stretching at increasing phantom elongations *ε*_ph_. The applicability of an elastic deformation model to match the experimentally obtained aspect ratios, *AR,* of prolate ellipsoids was evaluated and deviations from the model were discussed.

## 2. Materials and Methods

### 2.1. Microparticle Preparation and Characterization

Microparticles were prepared from different PPDL-PTHF materials, which are multiblock copolymers synthesized from precursors of oligo(ω-pentadecalactone) and oligotetrahydrofuran, as well as from PPDL-PCL and PCL-PCL multiblock copolymers comprising oligo(ε-caprolactone) segments (for synthesis and characterization, see Supplementary Materials). Particle preparation involved to dissolve 40 or 80 mg of the respective polymer in 1 mL of methylene chloride, dispersing the polymer solution by vortexing it (2500 rpm; MS1, IKA, Staufen, Germany) in a 2 mL aqueous solution of 2 wt.% polyvinyl alcohol (PVA; Mowiol^®^ 4-88; Kuraray, Frankfurt a.M., Germany), and adding it to a hardening bath (0.5 wt.% Mowiol^®^ 4-88) for solvent extraction/evaporation with magnetic stirring for 3 h. The microparticles were collected by centrifugation and lyophilized at 0.080 mbar (Alpha 1-2LD plus, Christ, Osterode, Germany). Analysis by static light scattering (Mastersizer 2000, Malvern, Herrenberg, Germany) using the Fraunhofer approximation showed average particle sizes of 30–50 µm.

### 2.2. Particle Embedding in PVA Phantoms, Stretching, and Microscopic Evaluation of Aspect Ratios

Programming of the particles was conducted by phantom stretching at *T*_high_ = 70 °C (Z1.0 with 200 N load cell, Zwick, Ulm, Germany), after incorporation of the particles in phantoms (films) prepared from aqueous solutions of 22.5 wt.% PVA (Mowiol^®^ 3-85) and 1 wt.% glycerol (casting with a 500 µm casting knife; dried at ambient conditions). Programmed particles were collected by dissolving the PVA film. Particle shapes were studied under a light microscope (Axio Imager.A1m, Carl Zeiss Microimaging, Göttingen, Germany) at room temperature. The dimensions of the particles were measured from images using the AxioVision software (version 4.6, Zeiss, Jena, Germany). The aspect ratio *AR* was calculated by dividing the longest particle diameter *x* by the shortest diameter *y,* as determined from the images.

### 2.3. Thermomechanical Tests

For determining the *E* modulus of the materials, film samples (50–100 µm thickness) were prepared from 22.5 wt.% aqueous solution of PVA (Mowiol^®^ 3-85), supplemented with 1 wt.% glycerol, or from 15 wt.% solutions of PPDL-PCL in chloroform, using a 0.5 mm casting knife. PPDL-PTHF films of 300–400 µm were prepared by melt compression. Samples were cut into dumbbell-shaped test specimens and *E* moduli were determined by tensile tests at 70 °C at a Zwick Z1.0 equipped with a thermochamber and a 200 N load cell (Zwick, Ulm, Germany). Films of the PCL-PCL multiblock polymer could not be tested, since the material is completely liquid at 70 °C due to the absence of hard chain segments.

### 2.4. Model Evaluation

As the acceptability criterion for the model, the coefficient of determination R^2^ was used as obtained by fitting the model (Equation (2), see Section 3.1) with the respective fixed *r* value to the mean of the experimental data. Curve fitting was conducted using the Levenberg–Marquardt algorithm in OriginPro software (2019 version, OriginLab Corporation, Northampton, MA, USA), allowing negligible alteration of *e* ± 0.01 to conduct the fit.

## 3. Results and Discussion

### 3.1. Deformation Models to Predict Aspect Ratios

**Figure 1:**
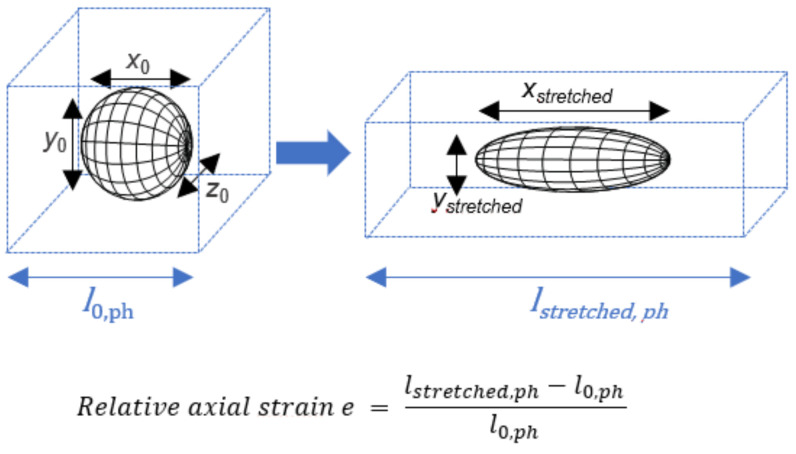
Scheme of the deformation of spherical particles to prolate ellipsoids (black grid) embedded in a phantom matrix (blue dashed box) by phantom stretching. A model for shape prediction should be suitable to describe the correlation of the phantom elongation *ε*_ph_ or relative axial strain *e*, and the particle dimensions, such as *x*_stretched_ or *AR,* after stretching.

The incorporation of particles of one polymeric material into a phantom matrix made from another polymeric material results in a composite system (Figure 1). If those materials show identical mechanical properties at the condition of deformation, they may be considered as a mechanically homogeneous elastic composite, meaning that the local flow of both polymeric materials (inclusion and phantom) occurs to an identical extent upon deformation. Based on this assumption and further considering the particles as incompressible materials of a constant volume, the stretching of a sphere with initial dimensions *x*_0_, *y*_0_ and *z*_0_ by a factor *f* in the *x*-direction should result in simultaneous thinning of the particle diameters in the *y*- and *z*-directions, according to Equation (1) (homogeneous composite material model) [26].

(1) $$AR=x\cdot y^{-1}=f\cdot f^{0.5},\;\;\;\;\;\;\;\;\;\;\;\;\;\;\;\;\;\;\;\;\;\;\;\mathrm{with}\;f=\frac{\varepsilon_{\mathrm{ph}}}{100\%}+1\;\;\;\mathrm{and}\;\;\;\varepsilon_{\mathrm{ph}}=\frac{l_{\mathrm{stretched},\mathrm{ph}}}{l_{0,\mathrm{ph}}}\cdot 100\%$$

where the particles are assumed to experience quantitative displacement matching *ε*_ph_ under the precondition that the *ε*_ph,local_ corresponds to the average overall *ε*_ph_ of the macroscopic phantom sample.

It is obvious that perfectly identical mechanical properties of the phantom matrix and embedded particles at the temperature of deformation will be present only in rare cases. More commonly, a mismatch of mechanical properties can be expected, leading to heterogeneous elastic composite materials. A model to predict the shape of particulate inclusions under such stretching conditions has been proposed [25], but has not applied so far to various materials to the best of the authors’ knowledge. This heterogeneous model is based on Eshelby’s concept of an elastic field with linear elastic particles embedded in an infinite linear elastic matrix. Again, incompressibility of the inclusion, continuity of displacement, and surface traction at the interface are assumed in this model (Equation (2); for details on the correct equation, see Supplementary Materials).

(2) $$AR=\frac{x}{y}=\frac{1+\left[\frac{1}{1-0.4\left(1-r\right)}\right]\cdot e}{1+\left[\frac{{\left(1+e\right)}^{-0.5}-1}{1-0.4\left(1-r\right)}\right]}$$

In Equation (2), *x* and *y* are the lengths of the major and minor particle axes, *e* is the relative axial strain (*e* = 1 means twice the initial length of the particle containing film), and *r* is the ratio of the *E* moduli of the particulate inclusion and the film matrix at the stretching temperature.

**Figure 2:**
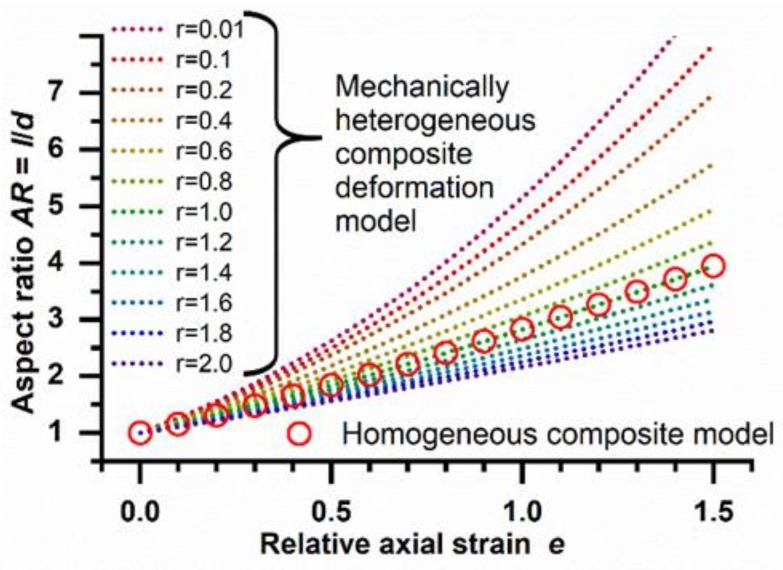
Graphical illustration of the impact of the ratio *r* of the *E* moduli of the particulate inclusion and the film matrix on the predicted *AR* of particles at different relative axial strains *e* (dotted lines; mechanically heterogeneous composite model according to Equation (2)). At *r* = 1, data match with the deformation model for a mechanically homogeneous composite (circles; see Equation (1)).

As shown in Figure 2, substantially different deformation patterns can be expected for different combinations of inclusions and matrices when applying the model for heterogeneous composite deformation (Equation (2)). Marked shifts of *AR* are proposed even upon only slight mismatches of particle and phantom mechanics (e.g., *r* = 0.8). At *r* = 1, i.e., identical *E* moduli of particulate inclusions and phantom matrix, the two models (Equations (1) and (2)) give consistent results of *AR* dependency on the axial strain.

### 3.2. Applicability of the Elastic Deformation Model to Multiblock Copolymer Microparticles

In order to evaluate the predictive power of the heterogeneous deformation model for sets of polymer particles, here, a series of PPDL-PTHF multiblock copolymers was selected with variations in the relative content of the two types of blocks. These materials undergo microphase separation into hard (PPDL) and soft (PTHF) domains, with the soft domains being molten at the temperature of deformation. The mechanical properties of the different materials were determined from film samples by tensile testing at 70 °C, which is the temperature that should later be applied for particle deformation. It is known that PPDL is semicrystalline at this condition [3], while the PTHF phases will be in the molten state [27]. By stepwise increasing the content of hard PPDL segments from 40 wt.% (PPDL_40_-PTHF) up to 60 wt.% (PPDL_60_-PTHF), the *E* modulus of the material could be systematically increased without altering the nature of the building blocks (Table 1). When set in relation to the *E* modulus of blank PVA phantoms, the ratios *r* of 0.33, 0.73, or 1.27, respectively, were calculated. Thus, the selected materials allowed to cover conditions in which the inclusions were slightly harder (PPDL_60_-PTHF, *r* = 1.27) and slightly softer (PPDL_40_-PTHF, PPDL_50_-PTHF) than the phantom matrix. Accordingly, a decreasing slope of the curves in the *AR–e* plots would be expected with increasing *r* (see Figure 2, Equation (2)).

**Figure 3:**
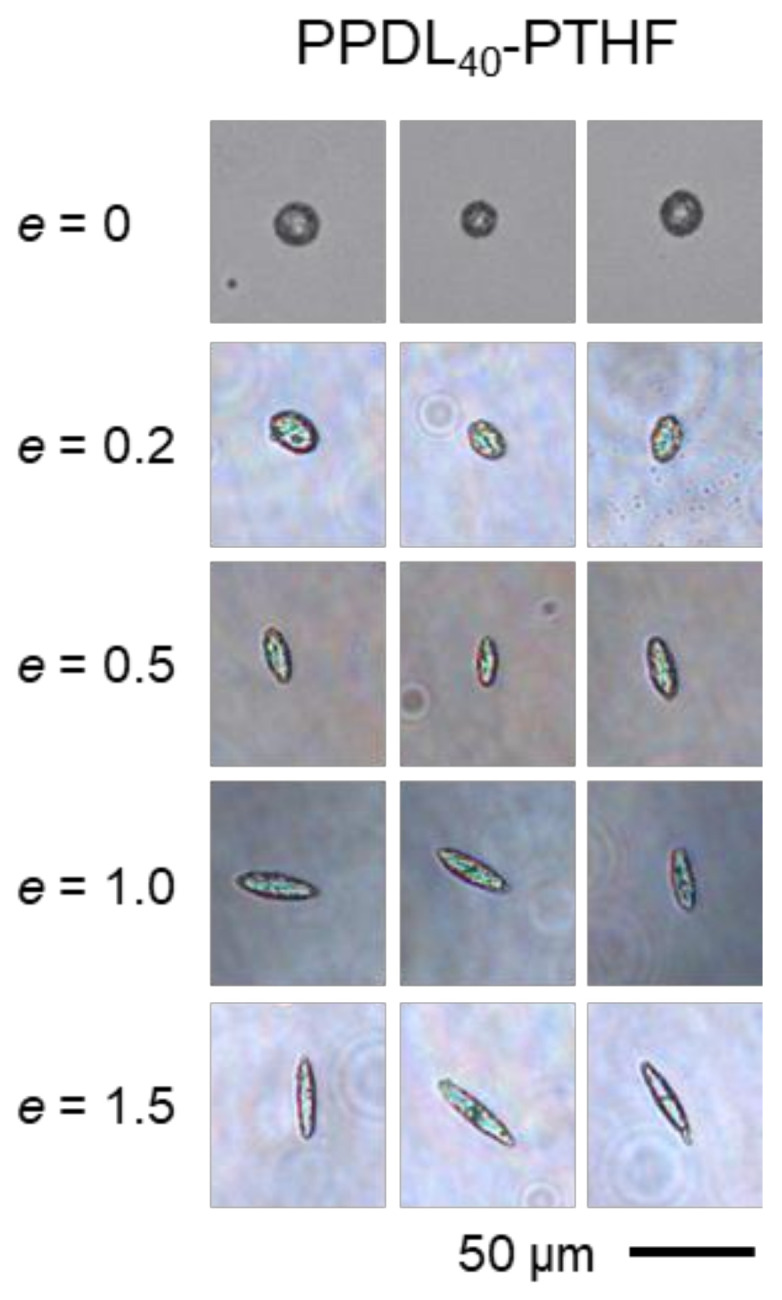
Exemplary light microscopy images for PPDL_40_-PTHF particles before and after deformation. Images were collected in aqueous suspension after isolation by phantom dissolution.

**Figure 4:**
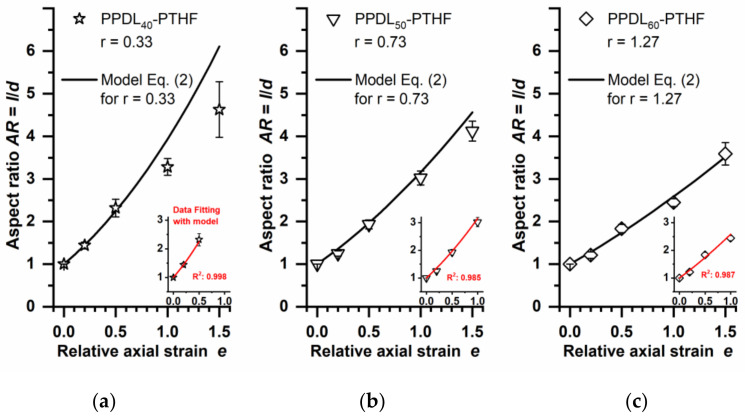
Correlation of experimentally determined *AR* and predictions of the heterogeneous deformation model according to Equation (2). Data for PPDL-PTHF particles after phantom stretching at different *e* (open symbols). Curve of the model as calculated based on the applicable *r* values (Table 1) for the respective PPDL-PTHF material (black lines). Insets: fitting of the model to experimental data up to *AR* values of ~3. (**a**) PPDL_40_-PTHF, (**b**) PPDL_50_-PTHF, and (**c**) PPDL_60_-PTHF. Mean and S.D. of *n* ≥ 20 particles for each condition. *R*^2^ coefficient of determination from curve fitting.

Microparticles of 30–50 µm were prepared from PPDL-PTHF by an emulsion-solvent evaporation technique, using PVA as a stabilizer during emulsification. The particles were freeze-dried and embedded in PVA phantoms, which were subjected to stretching at *ε*_ph_ = 20, 50, 100, or 150% (*e* = 0.2, 0.5, 1.0, or 1.5). The particles’ aspect ratios were microscopically evaluated after dissolution of the PVA phantoms (Figure 3). Experimental *AR* values of the isolated PPDL_60_-PTHF particles (*r* = 1.27) corresponded well to the prediction of Equation (2) even for the highest tested *e* = 1.5 (Figure 4C). For softer particles at intermediate *r* = 0.73, it appeared that the experimental values deviated from the model at high *e* = 1.5. It is also interesting to note that the softest particles made of PPDL_40_-PTHF (*r* = 0.33) showed a substantial deviation from the model at *e* ≥ 1.0, i.e., they deformed to a lower extent than expected, despite being softer than the phantom matrix. However, *AR* > 4 could still be observed for those PPDL_40_-PTHF particles, which was higher than for harder particles (PPDL_50_-PTHF, PPDL_60_-PTHF) of the same material class under identical deformation conditions. To verify the model further, curve fitting was performed, showing excellent *R*^2^ values (Figure 4 insets) for the respective experimental data up to an AR of ~3.

### 3.3. Investigation of Limitations of the Deformation Model for the Multiblock Copolymers

Considering the preconditions of the deformation model, a number of potential causes of the observed deviations of experimental data and the model may need to be reviewed. While the assumed incompressibility of the inclusions can be considered to apply for the selected particles at the given experimental conditions, the continuity of displacement and surface traction at the interface may be less clear and should be checked. Theoretically, voids may form in the PVA phantoms in case of poor adhesive forces between the phantom matrix and inclusion [28], resulting in a missing transfer of mechanical stress to the inclusion. This hypothetical phenomenon should be most expressed for the hardest particles (PPDL_60_-PTHF) rather than softest particles. A microscopic evaluation of the stretched phantoms with PPDL_60_-PTHF particles did not show any voids around the particles, even at high *e* = 1.5, thus allowing us to exclude poor adhesion issues at particle-phantom interphases. 

Another key assumption of the model is that both the phantom and the inclusion are made from elastic materials. In a recent study, it was shown that the PVA phantoms showed elastic deformation and integrity over a wide deformation range, with a tendency of necking and breakage only at very high elongations (*ε*_ph_ of 250%) [29], which is much higher than the *ε*_ph_ up to 150% applied here. Furthermore, it was observed that the phantoms showed excellent shape fixity of ~100% after cooling and release of the stress as applied for phantom deformation [29], confirming that there was no elastic recovery of the phantom after stretching that could hypothetically have caused a lower *AR* of the particulate inclusions. This leads to the assumption that the material of the particulate inclusions, rather than the characteristics of the phantom or the phantom-particle interface, are restricting the achievable particle deformations, particularly at a high content of soft PTHF domains. 

In fact, considering the phase separation of the PPDL-PTHF materials that contain molten (PTHF) and some crystalline domains (PPDL) at *T*_stretch_ = 70 °C, at least a certain portion of the particle volume (PPDL crystallites) should not behave as an elastic body during stretching deformation. Again, if the presence of hard PPDL domains caused the deviations from the deformation model, this phenomenon should be strongest at high content (PPDL_60_-PTHF) rather than at low content of PPDL (PPDL_40_-PTHF). As the opposite pattern was observed, it was concluded that the behaviour of the soft phase, rather than the hard phase, requires further investigation. Specifically, the authors assumed that the entanglements of polymer chains in the soft domains were the main cause of the restrictions of achievable *AR* and became effective at a certain *AR* that may be characteristic for the specific material.

**Figure 5:**
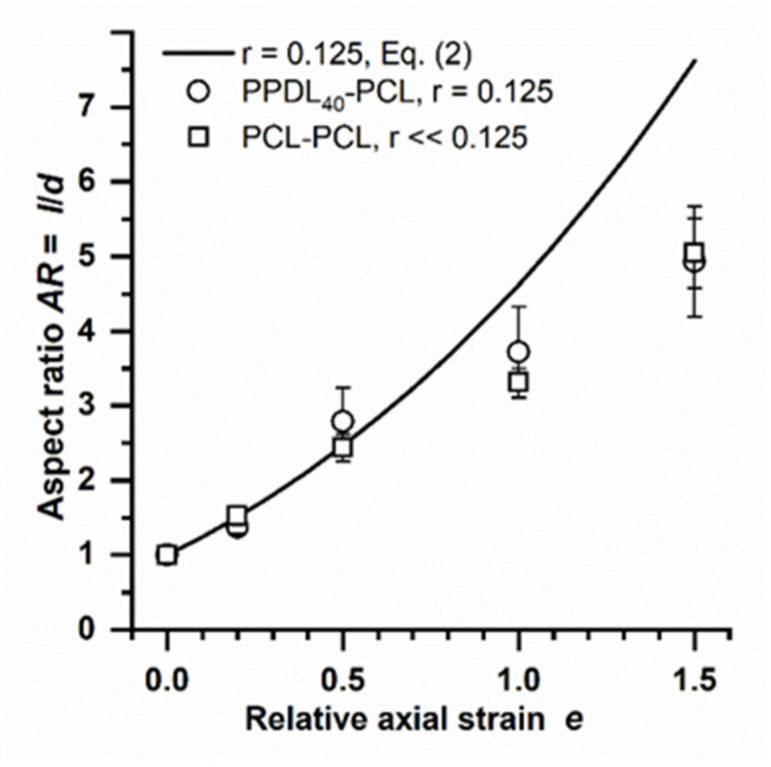
Experimental AR data of PPDL_40_-PCL and PCL-PCL in comparison to the deformation model (Equation (2)). Mean and S.D. of *n* ≥ 20 particles.

In order to test this hypothesis, alternative multiblock copolymers, PPDL_40_-PCL and PCL-PCL, were investigated. PPDL_40_-PCL was selected because of its lower *E* modulus compared to PPDL_40_-PTHF (Table 1). The corresponding calculated *r* = 0.125 of PPDL_40_-PCL particles incorporated in PVA phantoms is roughly half of the value of PPDL_40_-PTHF; thus, even higher *AR* should be achieved at a given *e*. When plotting the predicted *AR* of the stretched PPDL_40_-PCL particles and experimental data, good agreement was found for *e* = 0.2 and 0.5, while deviations in these very soft particles from the model were again observed at higher *e* ≥ 1 (Figure 5). The PCL-PCL multiblock copolymer used here as a control material does not contain segments to build hard domains, is fully molten at 70 °C (no *E* modulus can be determined by tensile testing), and can be estimated to show an even further reduced *r* (here stated as *r* << 0.125). Interestingly, the PCL-PCL multiblock polymer particles showed an identical *AR* pattern to PPDL_40_-PCL particles (Figure 5).

Overall, these investigations suggest that deviations from the model (Equation (2)) at certain *AR* values are not influenced by the absence or presence of crystalline domains. Instead, these observations support the conclusion that entanglements of polymer chains in the molten phase act as physical netpoints, limiting the range of elastic deformability, and thus cause deviations from the elastic deformation model above a certain *e*. Importantly, this does not diminish the value of the deformation model as it indicates when the elastic deformation range of particles has been exceeded. For the multiblock copolymers studied here, the model appears to be a practical approach for *AR* predictions up to the range of *AR*~3–4.

When aiming to transfer these findings to other particle materials, proper selection of *T*_stretch_ is needed to allow for chain mobility either in the entire particle volume or in phase-separated domains. An initial screening of the temperature dependency of *E* moduli of phantom and particle materials by tensile testing (film samples) should be conducted to determine the reasonable conditions, e.g., with *r* being in the range of 0.1–1.5. As illustrated in Figure 2, for much softer and much harder particles, changes in *r* will be less effective to change *AR*.

## 4. Conclusions

The elastic deformation model for mechanically heterogeneous composites can be used to precisely predict the achievable *AR* of prolate ellipsoids prepared by the phantom stretching method within certain boundaries. While entanglements of polymer chains represent a natural limitation to elastic deformation, the model could be successfully applied to PPDL-PTHF multiblock copolymer particles of different mechanical properties, adapting the *AR* values up to ~3–4. The authors are confident that the deformation model can be transferred to other particle materials based on the screening of temperature dependency of mechanical properties and selecting stretching temperatures that correspond to a reasonable ratio of the mechanical properties of phantom and particle inclusion.

## Figures and Tables

**Table 1:** Structure and properties of investigated polymers.

Sample	*E* at 70 °C ^1^ (MPa)	*r* = *E*_Inclusion_/*E*_Phantom_
*Multiblock copolymers*		
PPDL_40_-PTHF	16	0.33
PPDL_50_-PTHF	35	0.73
PPDL_60_-PTHF	61	1.27
PPDL_40_-PCL	6	0.125
PCL-PCL	− ^2^	− ^2^
*Phantom matrix*		
PVA	48	n.a.

^1^ The *E* moduli were determined from film samples in tensile tests. ^2^ For the PCL-PCL multiblock polymer, the mechanical properties could not be determined at 70 °C by tensile testing, since the polymer was completely molten. n.a. = not applicable.

## Data Availability

The data presented in this study are available upon request from the corresponding author.

## Supplementary Materials

The following supporting information can be downloaded at: https://www.mdpi.com/article/10.3390/polym14183762/s1, Description of: Deformation model for mechanically heterogeneous composites; Figure S1: Exemplary comparison of shapes of *AR–e* curves; Description of polymer synthesis; Table S1: Structure and properties of investigated polymers, including molecular weights, segment composition, thermal properties and mechanical properties. Refs. [3,25,30,31,32] are cited in the SI file.
