# Supplementary material for: Predictive Shapes of Ellipsoid PPDL-PTHF Copolymer Particles Prepared by the Phantom Stretching Technique
Source: Polymers (Basel). 2022 Sep 8;14(18):3762. doi: 10.3390/polym14183762 (PMC9502769; doi:10.3390/polym14183762)
Supplement: Supplementary file 1 [file polymers-14-03762-s001.zip › polymers-1859843-supplementary.pdf]

Supplementary Information

Predictive Shapes of Ellipsoid PPDL-PTHF Copolymer Particles Prepared by the Phantom Stretching Technique

Christian Wischke [*,†] and Dieter Hofmann

Institute of Active Polymers, Helmholtz-Zentrum Hereon, Kantstrasse 55, 14513 Teltow, Germany

* Correspondence: christian.wischke@hereon.de or christian.wischke@pharmazie.uni-halle.de

[†] Current address: Institute of Pharmacy, Martin-Luther University Halle-Wittenberg, 06099 Halle, Germany.

Deformation model for mechanically heterogeneous composites

The deformation model investigated here has been published by Keville et al. in 1991 [1] as presented in Supp. Eq. 1.

$$AR = \frac{x}{y} = \frac{1+\left[\frac{1}{1-0.4(1-r)}\right]^{e}}{1+\left[\frac{(1+e)^{0.5}-1}{1-0.4(1-r)}\right]} \qquad \text{Supp. Eq. 1, as published in [1]}$$

This version of the equation has been reprinted in later publications [2] [3], but has not been practically used by others to the authors knowledge.

The authors noted that the equation cannot be used as reported in [1]. When applying Supp. Eq. 1 to a particulate inclusion that is clearly softer than the matrix ($r = 0.5$) and increasing e, a relationship as plotted in Supp. Fig. 1 is obtained. The curve suggests an unrealistic aspect ratio AR of spheres being $AR = 2$ at $e = 0$ (no stretching), which surprisingly decreases with increasing e.

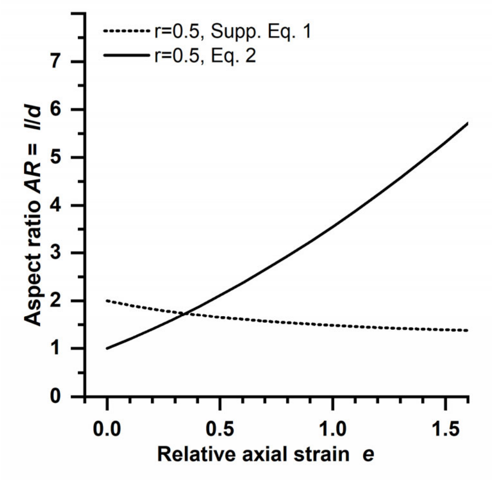

Figure S1. Exemplary comparison of shapes of $AR - e$ curves for r= 0.5 when either using Supp. Eq. 1 [1] or Eq. 2.

The authors systematically evaluated all variables in the equation. By correcting the position of e in numerator and the sign "+0.5" to "–0.5" in the denominator, Eq. 2 as reported in the main manuscript has been derived.

$$AR = \frac{x}{y} = \frac{1+\left[\frac{1}{1-0.4(1-r)}\right]\cdot e}{1+\left[\frac{(1+e)^{-0.5}-1}{1-0.4(1-r)}\right]} \qquad \text{Eq. 2 (see main manuscript)}$$

With this correct version (Eq. 2) of the model proposed by Keville, which could be confirmed in the archives of Purdue University [4], reasonable correlations of AR and e can be predicted (Figure S1).

Polymer synthesis

PPDL-PTHF multiblock copolymers were synthesized from diol precursors of oligo(ω-pentadecalactone)diol (OPDL; number average molecular weight M_n 3 kDa) and oligo(tetrahydrofuran)diol (OTHF; M_n 3 kDa) at weight ratios of 40/60, 50/50, and 60/40. OPDL, OTHF, and hexamethylene diisocyanate were dissolved in chloroform at 60 °C and

polymerized for 18 h using dibutyltin dilaurate as catalyst. After precipitation and washing with *n*-hexane, the obtained PPDL-PTHF materials were vacuum dried and characterized. The molecular weights were determined by GPC with universal calibration as shown in Supp. Tab. 1. The indices in polymer labelling (e.g. $PPDL_{40}PTHF$) indicate the weight ratios of the PPDL blocks.

The $PPDL_{40}$-PCL multiblock copolymer was synthesized from vacuum dried linear polyester diols, i.e., 40 wt.% oligo(ω-pentadecalactone)diol [OPDL; number average molecular weight M_n 3 kDa; synthesized by ring opening polymerization] and 60 wt.% oligo(ε-caprolactone)diol [OCL; Capa 2304, Perstorp, Cheshire, UK] by reaction with hexamethylene diisocyanate at 85 °C in dimethyl carbonate using dibutyltin dilaurate as catalyst. The material was subjected to subsequent purification and drying. The results of GPC characterization with universal calibration are shown in Supp. Tab. 1.

In a similar manner, a PCL-PCL multiblock polymer was synthesized from oligo(ε-caprolactone)diol (M_n 2 kDa) by reaction with hexamethylene diisocyanate and subsequent purification. The results of GPC characterization with universal calibration are shown in Table S1.

Table S1. Structure and properties of investigated polymers.

Sample	M_n (kDa)	PD	Hard segment M_n (kDa)	Hard segment (wt.%)	Soft segment M_n (kDa)	Thermal properties T_g (°C)	$T_{m,1}$ (°C)	$T_{m,2}$ (°C)	E at 70 °C [1] (MPa)	$r = \frac{E_{Inclusion}}{E_{Phantom}}$
Multiblock copolymers										
$PPDL_{40}$–PTHF	26	1.8	5	40	3	n.d.	21 (PTHF)	89 (PPDL)	16	0.33
$PPDL_{50}$–PTHF	35	2.3	5	50	3	n.d.	11 (PTHF)	89 (PPDL)	35	0.73
$PPDL_{60}$–PTHF	26	2.1	5	60	3	n.d.	9 (PTHF)	89 (PPDL)	61	1.27
$PPDL_{40}$–PCL	39	2.3	3	40	3	- 57	48 (PCL)	83 (PPDL)	6	0.125
PCL-PCL	13	1.5	n.a.	n.a.	2	n.a.	44 (PCL)	–	– [2]	– [2]
Phantom matrix										
PVA	5.6 [3]	2.5 [3]	–	–	–	56	n.a.	n.a.	48	n.a.

[1] The E modulus was determined from film samples in tensile tests. [2] For the PCL-PCL multiblock copolymer, the mechanical properties could not be determined at 70 °C by tensile testing since the polymer was completely molten. [3] According to [5]. n.a. = not analysed, n.d. not detected

Supplementary references

[1] K. M. Keville, E. I. Franses, J. M. Caruthers, *J. Colloid Interface Sci.* **1991**, *144*, 103.
[2] Y. Lu, Y. D. Yin, Y. N. Xia, *Adv. Mater.* **2001**, *13*, 271.
[3] Y. Lu, Y. D. Yin, Z. Y. Li, Y. N. Xia, *Langmuir* **2002**, *18*, 7722.
[4] K. M. Keville-Polizopoulus, "Preparation, characterization, and flow behavior of dispersions of monodisperse microspheroids", Prudue University, 1988.
[5] C. Wischke, M. Schossig, A. Lendlein, *Small* **2014**, *10*, 83.
